# Design and Evaluation of Orally Dispersible Tablets Containing Amlodipine Inclusion Complexes in Hydroxypropyl-β-cyclodextrin and Methyl-β-cyclodextrin

**DOI:** 10.3390/ma15155217

**Published:** 2022-07-28

**Authors:** Marian Novac, Adina Magdalena Musuc, Emma Adriana Ozon, Iulian Sarbu, Mirela Adriana Mitu, Adriana Rusu, Simona Petrescu, Irina Atkinson, Daniela Gheorghe, Dumitru Lupuliasa

**Affiliations:** 1 Department of Pharmaceutical Technology and Biopharmacy, Faculty of Pharmacy, “Carol Davila” University of Medicine and Pharmacy, 6 Traian Vuia Street, 020945 Bucharest, Romania; marian.novac@rez.umfcd.ro (M.N.); dumitru.lupuliasa@umfcd.ro (D.L.); 2 “Ilie Murgulescu” Institute of Physical Chemistry, 202 Spl. Independentei, 060021 Bucharest, Romania; arusu@icf.ro (A.R.); simon_pet@icf.ro (S.P.); iatkinson@icf.ro (I.A.); chiscan_danny@icf.ro (D.G.); 3 Department of Pharmaceutical Physics and Biophysics, Drug Industry and Pharmaceutical Biotechnologies, Faculty of Pharmacy, “Titu Maiorescu” University, 004051 Bucharest, Romania

**Keywords:** amlodipine besylate, hydroxypropyl-β-cyclodextrin, methyl-β-cyclodextrin, lyophilization technique

## Abstract

The development of new orally dispersible tablets containing amlodipine (AML) inclusion complexes in hydroxypropyl-β-cyclodextrin (HP-β-CD) and in methyl-β-cyclodextrin (Me-β-CD) was studied. The methods of obtaining amlodipine and the physical and chemical properties of the inclusion complexes using the two cyclodextrins was investigated separately. Solid inclusion complexes were obtained by three methods: kneading, coprecipitation, and lyophilization, at a molar ratio of 1:1. For comparison, a physical mixture in the same molar ratio was prepared. The aim of the complexation process was to improve the drug solubility. As the lyophilization method leads to a complete inclusion of the drug in the guest molecule cavity, for both used cyclodextrins, these types of compounds were selected as active ingredients for the design of orally dispersible tablets. Subsequently, the formulation of the orodispersible tablets containing AML-HP-β-CD and AML-Me-β-CD inclusion complexes and quality parameters of the final formulation were evaluated. The results prove that F1 and F4 formulations, based on silicified microcrystalline cellulose, which contains insignificant proportions of very small or very large particles, had the lowest moisture degree (3.52% for F1 and 4.03% for F4). All of these demonstrate their porous structure, which led to good flowability and compressibility performances. F1 and F4 formulations were found to be better to manufacture orally dispersible tablets.

## 1. Introduction

The dissolution rate and, in consequence, the solubility of a drug, is one of the most important factors that determines the desired pharmacological response. Generally, the low aqueous solubility of drugs represents a major challenge regarding the development of a pharmaceutical formulation. Regarding the improvement of the solubility of drugs, and therefore their oral bio-availability, numerous approaches are reported in the literature: physical (such as drug dispersion in carriers, cryogenic techniques, particle size reduction, modification of the crystal habit) or chemical (such as derivatization, change in pH, complexation, derivatization, salt formation) modifications and miscellaneous (such as the supercritical fluid process, the use of surfactants, hydrotrophy) methods [1]. The selection of an adequate technique is dependent on (i) the properties of drugs (chemical nature, melting point, physical nature, pharmacokinetic behavior, regulatory requirements), (ii) the nature of the selected excipients, and (iii) the nature of the considered dosage form. Among all available techniques able to enhance drug solubility, dissolution rate, and bioavailability, the inclusion complex method was the most widely used one [2]. The inclusion process takes place when a nonpolar region/molecule, named the “guest” molecule, is embedded into the cavity of a “host” molecule. Cyclodextrins are cyclic oligosaccharides produced by enzymatic degradation of starch, which are composed of several α-1,4-linked glucopyranose units [3,4]. Due to their hydrophobic internal part and the hydrophilic outer surface, they are the most common ones used as “guest molecules.” Their ability to form inclusion complexes with many drugs is useful to increase the drug solubility, stability, and bioavailability [5,6]. Usually, inclusion complexes are formed by partial or total inclusion of the guest molecule in the CD cavities in different molar ratios.

Amlodipine is a blocker of calcium channels from the group of dihydropyridine compounds, commonly used in the treatment of hypertension and angina pectoris [7,8,9]. Two forms of amlodipine are reported: S-enantiomer, which is more active, and R-enantiomer [10,11]. Amlodipine besylate (AML), the besylate salt of amlodipine, has become the most used blood pressure medication [12] and the most widespread cardiovascular drug [13]. The different polymorph forms of amlodipine besylate (hydrated or solvated forms) can affect pharmaceutical properties like morphology, stability, solubility, dissolution rate, and also the final performance of solid dosage tablets. Amlodipine has a slower absorption rate, is slightly soluble in water, and was classified according to the Biopharmaceutics Classification System (BCS) provided by the U.S. Food and Drug Administration in Class I (high solubility and high permeability) [14]. To enhance the solubility and the dissolution rate of the amlodipine and, subsequently, its therapeutic effect, different studies were performed [15,16,17,18,19,20,21,22]. To improve its adsorption performance from oral solid dosage form, complexation with cyclodextrins is a promising method [23,24,25]. Many studies were devoted to the various formulations of amlodipine with cyclodextrins in order to improve its low solubility. Mielcarek et al. demonstrated that the solubility of amlodipine in the clathrates obtained by kneading and lyophylization methods was three times greater than in the crystalline form [24]. Bhardwaj et al. [26] prepared fast oral dissolving tablets of amlodipine by the sublimation method and direct compression, and using different concentrations (2%, 4% and 6%) of superdisintegrants, such as croscarmellose (Ac-Di-Sol), crospovidone (Kollidon-CL), and sodium starch glycolate. Camphor was used as a sublimating agent. Among all three disintegrants, croscarmellose in 6% concentration was found to have faster dissolution by causing a disintegration of the tablets within 11 s. A combination of both sodium starch glycolate and crospovidone superdisintegrants in concentrations of 5–10% were found to be better to manufacture fast-dissolving tablets of amlodipine besylate, which showed the lowest disintegrating time and a faster dissolution of 87% [27]. By using crospovidone as a superdisintegrant, solid dispersion of amlodipine tablets manufactured in a 1:4 ratio showed an increased solubility and dissolution rate [15]. Using natural superdisintegrants, such as fenugreek seed mucilage and Ocimum basilicum (basil) gum in different concentrations (2–10 mg), the formulations prepared by the direct compression method with the highest amount of the disintegrant possessed the fastest disintegration time and higher stability (for three months) [28].

The objectives of this work were (i) the preparation of inclusion complexes, in a 1:1 molar ratio, of amlodipine besylate with hydroxypropyl-β-cyclodextrin and amlodipine besylate with methyl-β-cyclodextrin in order to improve its dissolution, and different synthesis procedures for different unit processes such as physical mixture, kneading, coprecipitation, and lyophilization were tried to prepare the inclusion complexes; (ii) the investigation, using several analytical techniques (Fourier transform infrared spectroscopy, scanning electron microscopy, X-ray diffraction analysis, and differential scanning calorimetry) of the formation of inclusion complexes; and (iii) to perform preformulation and formulation studies for orodispersible tablets containing AML-HP-β-CD and AML-Me-β-CD inclusion complexes. The novelty of the present study consists of the obtaining of orally dispersible tablets containing amlodipine inclusion complexes in hydroxypropyl-β-cyclodextrin and in methyl-β-cyclodextrin as final products. Orally dispersible tablets are a special type of tablet, described by European Pharmacopoeia as uncoated tablets that are placed in the mouth, where they are readily dispersed within 3 min before swallowing. The oral route of administration of a drug represents a low-cost therapy, due to its versatility, convenience, simplicity, and most widespread patient acceptability. Such orally dispersible tablets were manufactured in the present work, and the physico-chemical and quality parameters of the final formulation were evaluated.

## 2. Materials and Methods

### 2.1. Materials

**Scheme 1:**
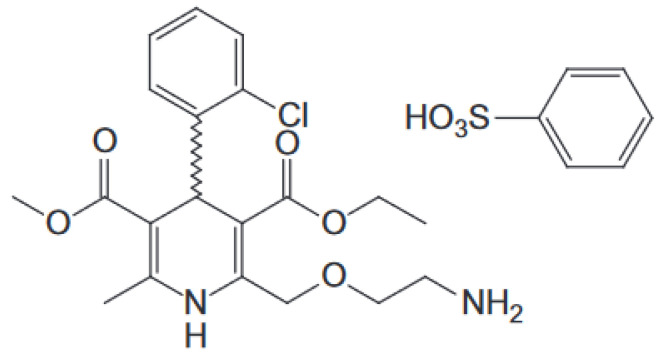
Chemical structure of amlodipine besylate (AML).

Amlodipine besylate (AML) (2-[(2-aminoetoxy)-methyl]-4-(2-chlorophenyl)-1,4-dihydro-6-methyl-3,5-pyridine dicarboxylic acid 3-ethyl 5-methyl esterbenzene sulfonate; Scheme 1), HP-β-CD, and Me-β-CD were purchased from Hong Kong Guokang Bio-Technology Co., Limited, Baoji City, Shaan’xi province, Mainland China. All other chemicals and solvents used in the study were of analytical reagent grade.

PROSOLV^®^ SMCC HD 90 and EXPLOTAB^®^ were provided by JRS PHARMA GmbH & Co. KG, Rosenberg, Germany, and Ludiflash^®^ was purchased from BASF, Ludwigshafen, Germany. F-MELT^®^ is produced by Fuji Chemical Industries Co., Ltd., Toyoma, Japan, and LIGAMED^®^ MF-2-V by Peter Graven NV, Venlo, The Netherlands.

A Mettler Toledo AT261 balance (with 0.01 mg sensitivity, Columbus, OH, USA) was used to weigh the materials.

### 2.2. Methods

#### 2.2.1. The Binary System Obtaining Processes

Several studies that investigated AML inclusion in different β-CDs cavities had already been performed and published, and they served as starting points for the present study. Lauro et al. [12] proved, with phase solubility studies, that the optimal molar ratio to form a stable complex is 2:1 drug/CD. Meantime, Malakzadeh et al. [29] calculated the formation constant and found that a 1:1 molar ratio leads to the encapsulation of AML in β-CDs. Considering the recorded results, a 1:1 AML:β-CDs molar ratio was selected for the analyzed complexes in the present research.

**Scheme 2:**
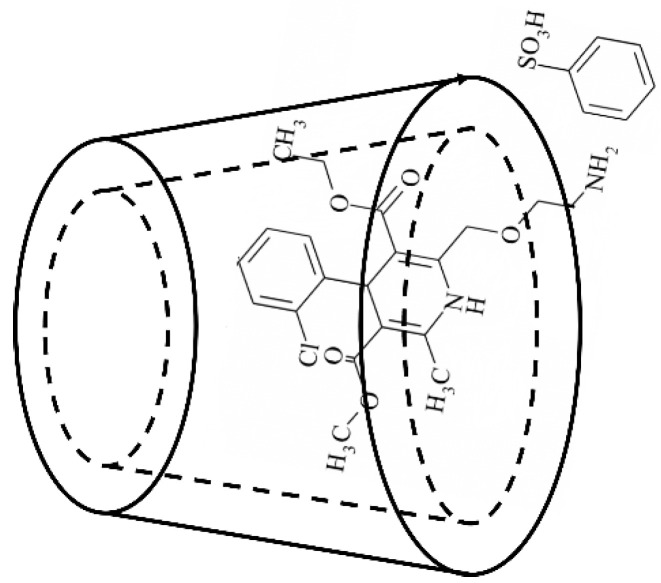
Schematic representation of the interaction between CDs and amlodipine besylate (1:1 molar ratio).

Both complexes (AML-HP-β-CD and AMPL-Me-β-CD) were prepared by different methods: kneading, coprecipitation, and lyophilization. In order to establish the most suitable technique that would lead to the complete inclusion of the drug, the binary systems were characterized by SEM, IR, DSC, and X-ray diffraction, in comparison to the raw materials and their simple physical mixtures [30,31] obtained in the same 1:1 molar ratio. A schematic representation of the interaction between CDs and amlodipine is shown in Scheme 2. According to literature data, the inclusion complexes of various drugs with different cyclodextrins is formed in a covalence manner, involving mainly hydrophobic interactions and van der Waals bonds, and frequently hydrogen bonds [29,32].

##### Kneading Product

Samples were weighed and wetted with a low amount (2 mL) of ethyl alcohol:water (50:50 *v*/*v*) mixture. Subsequently, the mixture was manually kneaded for about 2 h at 21 °C in a ceramic mortar. The obtained consolidated material was sieved through a 12-mesh sieve and the collected granules were dried at temperature of 25 °C for 24 h. Finally, the material was ground until a fine powder was achieved.

##### Preparation of the Inclusion Compounds Using Liquid-State Coprecipitation and Lyophilization Techniques

Accurate weighed quantities of AML and the two beta-cyclodextrin derivatives were dissolved in ethanol and distilled water, respectively. The aqueous solution of cyclodextrin compounds was slowly added to the alcoholic solution of amlodipine. Next, the two obtained mixtures were stirred for 6 h at room temperature at 750 rpm in a magnetic stirrer (Heidolph MR 3001 K apparatus, Schwabach, Germany).

Subsequently, the coprecipitated products were obtained using the following procedure: The obtained mixtures from the magnetic stirrer were filtered, and then the sediments were dried at a temperature of 25 °C for 24 h.

In the lyophilization technique, the resultant stirred clear solutions were frozen, then the frozen solutions were lyophilized in a freeze-dryer (Christ ALPHA 1–2, manufactured by B Braun Biotech International, Melsungen, Germany) lyophilizer at −60 °C for 12 h. The obtained powders were protected in a desiccator.

##### Preparation of Physical Mixtures

The substances, weighed according to the 1:1 molar ratio, were mixed in a mortar for 5 min at room temperature.

#### 2.2.2. Physicochemical Characterization

Fourier transform infrared spectra of raw materials, the physical mixture, and inclusion complexes were achieved with a NICOLET 6700 FT-IR spectrophotometer from Thermo Electron Corporation (Waltham, MA, USA). Spectra acquisitions were achieved from KBr pellets over the range of 4000–500 cm^−1^ in transmittance mode.

X-ray powder diffraction pattern of raw materials, the physical mixture, and products of the inclusion process were obtained with a Rigaku Ultima IV diffractometer (Tokyo, Japan) system equipped with a CuKα radiation at λ = 1.5406 Å in parallel beam geometry. The parameters of the process were set to a step size of 0.02° and speed of 2°/min. The diffractograms were recorded in the 2θ angle range between 5° and 60°.

The surface morphology was analyzed with a scanning electron microscope FEI Quanta 3D FEG (Brno, Czech Republic). The dry samples were fixed on a double-adhesive carbon-coated tape. The scanning analysis was conducted without coating. The secondary electron micrographs were recorded at accelerating voltage in the range of 5–20 kV in high vacuum mode.

Differential power compensated calorimetry (DSC; DSC-8500 Perkin-Elmer, Shelton, CT, USA) with a cooling system (Intracooler III) was used to analyze the thermal changes. Analyses were performed in sealed aluminum pans (with a volume of 20 μL) under a nitrogen atmosphere at a flow rate of 20 mL min^−1^ in the 30–250 °C range, with a heating rate of 10 °C min^−1^. An isothermal step at 30 °C for 2 min was initially maintained. The apparatus calibration was carried out using a standard with a known melting temperature and heat of fusion (indium, *T*_fus_ = 156.7 °C; Δ*H*_fus_ = 28.5 J g^−1^). The DSC curves normalized to sample weight were used to calculate the thermal effects with Pyris Software for Windows (version 13.4.0.0040).

#### 2.2.3. Preformulation and Formulation Studies for Orodispersible Tablets Containing AML-HP-β-CD and AML-Me-β-CD Inclusion Complexes

As the physico-chemical studies performed on the inclusion complexes revealed that the lyophilization technique led to the complete encapsulation of AML in the CDs cavity, these types of systems were selected as active ingredients for the orally dispersible tablets.

The lyophilized complexes present an amorphous character, and it is well known that although it is beneficial for the dissolution performance, it induces low flowability and compressibility properties. These attributes are essential for the manufacturing of tablets, especially for the direct compression process. In order to avoid the use of high temperatures and moisture, direct compression is preferable to compression after wet granulation. However, processing the lyophilization powder is challenging and needs proper formulation and process parameters. The selection of the excipients is a key step in the development of the tablets and achieving a suitable flowability and compressibility of the compression blend is crucial for ensuring high-quality manufacturing of solid dosage forms.

##### Formulation of Direct Compression Blends

Different direct compression excipients suitable for oral dispersion were selected and the amounts of inclusion complexes and auxiliary materials were established in order to ensure a 5 mg AML dosage for each tablet.

Table 1 shows the blend formulations. F1–F3 contained the AML-HP-β-CD inclusion complex and F4–F6 contained the AML-Me-β-CD inclusion complex.

Three co-processed materials (PROSOLV^®^ SMCC HD 90, F-MELT^®^, and Ludiflash^®^) were selected as major excipients in different formulations, and the characteristics of the complex powders and final tablets were determined in comparison.

PROSOLV^®^ SMCC HD 90 is silicified microcrystalline cellulose; F-MELT^®^ is a spray-dried excipient that contains saccharides, a disintegrating agent, and an inorganic excipient; and Ludiflash^®^ consists of D-mannitol, crospovidone, and a polymer dispersion based on polyvinyl acetate [33].

Sodium starch glycolate acts as a superdisintegrant, and magnesium stearate is needed for its lubricant properties.

##### Materials for Direct Compression Preparation

The accurate weights of materials were sieved (using a 20-mesh sieve) and mixed at room temperature for 20 min at 30 rpm speed in a CMP 12 Plexiglas cube blender (Pharmag GmbH, Klipphausen, Germany).

##### The Pharmacotechnical Properties of the Direct Compression Powders

Six determinations of each material were performed.

The flowability measurements were conducted using an Automated Powder and Granulate Testing System PTG-S3, (Pharma Test Apparatebau GmbH, Hainburg, Germany). Methodology: 60 g of each sample were passed through an orifice with a standardized diameter, and the flow time and rate and the response angle were measured. A CISA Sieve Shaker Mod. RP 10 (Cisa Cedaceria Industrial, Barcelona, Spain) was used for the particle size distribution determination. A total of 50 g of each sample was passed through a vibrating standard sieve arranged in ascending degrees of coarseness and shaken for 20 min. The sample retained on each sieve was collected and then weighed. A Mettler Toledo HR 73 halogen humidity analyzer (Mettler-Toledo GmbH, Greifensee, Greifensee, Switzerland) was used for determining the moisture content by measuring the loss of mass of the sample on drying. A Vankel Tap Density Tester (Vankel Industries Inc., Atlanta, GA, USA) was used to measure the compressibility. A total of 50 g of each sample was transferred to a graduated cylinder and then the bulk volume was read. Subsequently, a specified number of mechanical taps was completed, and the tapped volume was then measured. The measurements of powder density (bulk and tap density) were used to determine the Hausner ratio HR, defined as the ratio between tapped and bulk density, and the Carr Index *CI* (Equation (1)):

(1) $$CI=\frac{100\left(\rho_{f}-\rho_{0}\right)}{\rho_{f}}$$

Powders with HR ≤ 1.25 and *CI* ≤ 10 are considered freely flowing and with a good compressibility [34].

#### 2.2.4. Development and Manufacturing of Orodispersible Tablets Containing AML-HP-β-CD and AML-Me-β-CD Inclusion Complexes

##### Formulation of the Orodispersible Tablets Containing AML-HP-β-CD and AML-Me-β-CD Inclusion Complexes

The pre-compression studies revealed that F1 and F4 materials, containing silicified microcrystalline cellulose, presented suitable characteristics for the direct compression process. It was decided to proceed with the compression on these two formulations, and in order to select the proper technological parameters needed to ensure high-quality tablets, the materials were compressed at different tableting forces (30 kN and 60 kN). The final formulations are represented in Table 2.

##### Manufacturing Process of Tablets for Oral Dispersion

A single-post eccentric Erweka EP-1 Tablet Press (Erweka, Germany) equipped with 10 mm flat punches was used to obtain tablets for oral dispersion employing two different compression forces of 30 kN and 60 kN. The final obtained tablets had an average weight of 350 mg, which corresponded to 5 mg AML per orally dispersible tablet.

#### 2.2.5. Quality Assessment Parameters

The obtained orally dispersible tablets were investigated in agreement with the compendial recommendations [35,36]. The tablets’ diameter and thickness were established using a VK 200 Tablet Hardness Tester (Vanderkamp, New York, NY, USA). The measured force necessary to initiate the fracture of a tablet was carried out using a VK 200 Tablet Hardness Tester for hardness determination. Friability studies were conducted using a Vankel friabilator at 25 rpm for 5 min. The weight loss in this process must be above 1% for good friability of the tablet. All measurements were performed for 10 tablets from each batch formulation. By separately weighing 20 tablets for each batch formulation, the average weight was determined [35].

##### *In Vitro* Disintegration Time

Disintegration time was established using the Erweka DT 3 apparatus (Erweka^®^ GmbH, Langen, Germany). Six tablets per product were examined in two media: in simulated saliva phosphate buffer at 37 ± 2 °C (pH = 6.8) and in distilled water at 37 ± 0.5 °C [35]. The disintegration completion time and disintegration onset time were observed and recorded for each individual tablet, with any residue of the unit remining on the apparatus screen.

##### *In Vitro* Dissolution Studies

Dissolution studies were performed utilizing the USP paddle apparatus (ERWEKA DT820 HH model, Heusenstamm, Germany) at a temperature of 37 ±  0.5 °C at a 75 rpm rotation speed. Six tablets of each batch formulation were examined in two dissolution media: (i) a compendial dissolution medium composed of 500 mL of 0.01 N HCl [8] and (ii) a biorelevant medium that simulated the composition of saliva: a mixture of 8g/L NaCl, 0.19 g/L KH_2_PO_4_ and 2.38 g/L Na_2_HPO_4_ at pH = 6.8 [37]. After 30 min, the amount of AML was dissolved in 2 mL dissolution medium. The determinations were carried out with a UV-VIS Nicolet Evolution 100 spectrometer at λ_max_ = 237 nm.

## 3. Results

### 3.1. Binary Systems Characterization

All binary systems obtained were white, uniform, and fine powders, with different appearances based on the preparation process.

### 3.2. Characterization of the Inclusion Complexes

**Figure 1:**
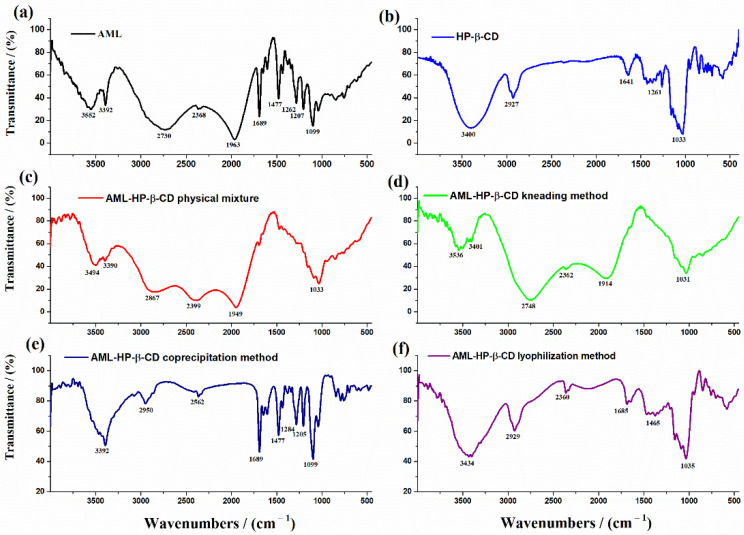
FTIR spectra of (**a**) AML, (**b**) HP-β-CD, (**c**) AML-HP-β-CD physical mixture, (**d**) AML-HP-β-CD kneading method, (**e**) AML-HP-β-CD coprecipitation method, and (**f**) AML-HP-β-CD lyophilization method.

In Figure 1 are represented the FTIR spectra of AML, HP-β-CD, the physical mixture and their inclusion complexes using the three methods of preparation: kneading, coprecipitation, and lyophilization methods. The FTIR spectrum of pure AML shows the presence of its main characteristic peaks at 3552 cm^−1^ and 3592 cm^−1^ due to the N-H amine stretching vibration; at 2730 cm^−1^ assigned to the aliphatic C-H stretch; at 1689 cm^−1^ due to ester carbonyl C=O stretches; at 1602 cm^−1^ and 1477 cm^−1^ assigned to aromatic skeletal stretches and CH_3_, CH_2_ bending, respectively; at 1262 cm^−1^ due to ester (C-O) asymmetric stretch; at 1207 cm^−1^ due to sulfonate (S=O) asymmetric stretch; at 1099 cm^−1^ due to aromatic C-Cl stretch; at 1039 cm^−1^ assigned to ether (C-O-C) symmetric stretch; and at 757 cm^−1^ due to aromatic (C-H) bending [38]. The FTIR spectrum of HP-β-CD showed characteristic bands that belonged to saccharides: 3400 cm^−1^ (O-H stretching vibration), 2927 cm^−1^ (C-H stretching vibration), 1641 cm^−1^ (O-H bending vibration), and 1033 (C-O bond vibration) [3,39,40]. The absorption peak found at 845 cm^−1^ is characteristic of an α-type glycosidic bond. The FTIR spectra of the AML-HP-β-CD physical mixture and the inclusion complexes obtained using coprecipitation method are comparable. The characteristic peaks of amlodipine were shifted to different frequencies (Figure 1). In the case of the compound obtained using the kneading and lyophilization processes there was no record of the bands whose position, shape, and intensity are characteristic for an amlodipine compound. In addition, comparing the FTIR spectrum of the HP-β-CD with the corresponding FTIR spectrum of the complexes with the lyophilization method, one can notice that the bands at 1685 cm^−1^ and 1465 cm^−1^ from the complex are not presented in the host molecule. This is evidence of the interaction of the drug molecule with the CD molecule through N-H groups [23]. In the inclusion complexes spectra, not only had the peaks shifted, but some of them also disappeared specifically when comparing the spectra of AML and pure CDs compounds and the obtained complexes. This is due to the entrapment of the drug into the cyclodextrin cavity by the formation of a hydrogen bond. Similar findings were found for other studied inclusion compounds [41,42].

**Figure 2:**
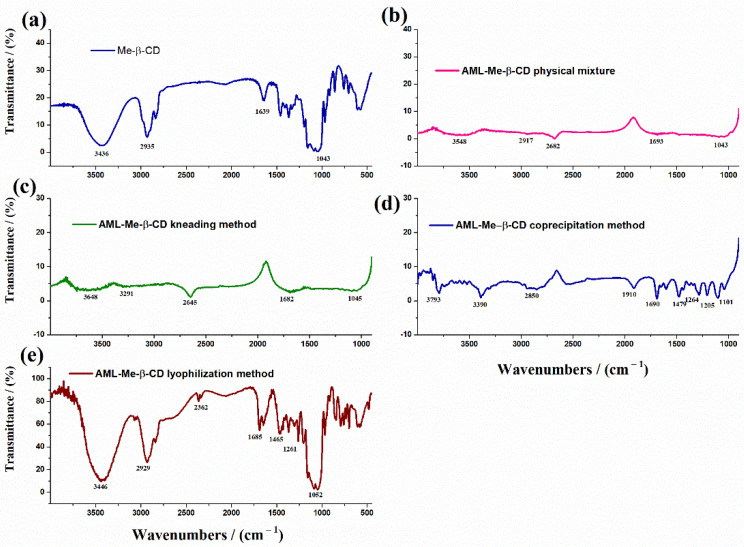
FTIR spectra of (**a**) Me-β-CD, (**b**) AML-Me-β-CD physical mixture, (**c**) AML-HP-β-CD kneading method, (**d**) AML-HP-β-CD coprecipitation method, and (**e**) AML-HP-β-CD lyophilization method.

In Figure 2 are represented the FTIR spectra of the Me-β-CD and AML-Me-β-CD physical mixtures and their inclusion complexes. The FTIR spectrum of Me-β-CD presented the main peak characteristics of saccharides as well as HP-β-CD, with some displacement. The peaks at 3436 cm^−1^ were due to the O–H stretching vibration, at 2935 cm^−1^ to the C–H stretching vibration, at 1639 cm^−1^ to the H–O–H bending vibration, and at 1043 cm^−1^ to the C–O bond vibration [3,43]. The changes observed in the FTIR spectrum of the AML-Me-β-CD physical mixture and inclusion complexes, such as the shift in the peaks or their reduction in intensity up to complete disappearance, depended on the preparation method. The disappearance of the characteristic peaks of the amlodipine and the presence only of Me-β-CD compound suggest the complete inclusion of the drug in the CD molecule in a molar ratio of 1:1. The FTIR spectra of the inclusion compound of AML and Me-β-CD obtained by kneading and the coprecipitation method were different compared with raw compounds, suggesting a different degree of interaction or amorphization in the different products [44].

**Figure 3:**
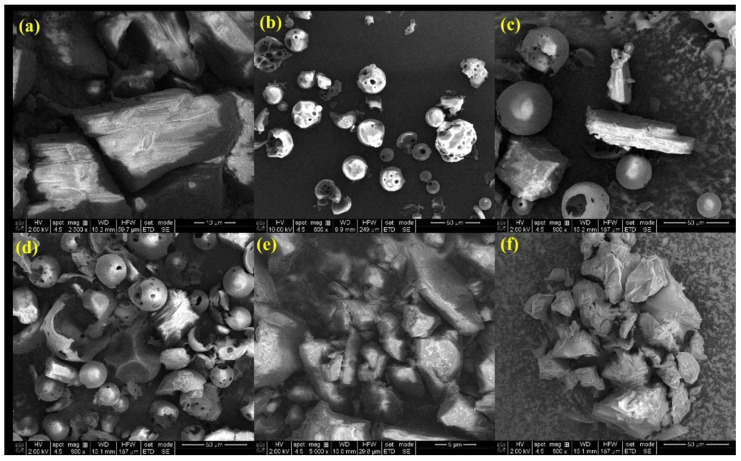
SEM images of (**a**) AML, (**b**) HP-β-CD, (**c**) AML-HP-β-CD physical mixture, (**d**) AML-HP-β-CD kneading method, (**e**) AML-HP-β-CD coprecipitation method, and (**f**) AML-HP-β-CD lyophilization method.

Scanning electron microscopy is a useful qualitative method for studying the structural characteristics of materials. This technique is not evidence of the formation of an inclusion compound, but it is helpful to assess the existence of a single component in the obtained formulation. SEM images obtained at different magnifications between 500× and 3000× were consistent at different parts of the sample. In the present study the most representative ones that provided significant information regarding the morphologies of studied samples were considered. The morphologies of AML, HP-β-CD, AML-HP-β-CD physical mixture, and the three compounds obtained using the kneading, coprecipitation, and lyophilization methods are represented in Figure 3. AML appeared as parallelogram-shape crystals (Figure 3a). For HP-β-Cd the spherical forms of particles were observed (Figure 3b). The SEM image of the physical mixture clearly shows the crystals characteristic of AML mixed with the HP-β-CD spherical particles (Figure 3c). This is evidence that in the physical mixture no inclusion complex was formed. In the system obtained by the kneading method, AML crystals adhering to the cyclodextrin surface were observed. In contrast, by using coprecipitation and lyophilization preparation methods, drastic changes were observed regarding the morphology of the obtained product. It is not possible in this case to differentiate the two component compounds. This is evidence of an interaction between the AML and the used cyclodextrin [44]. If in the case of the coprecipitation compound it was still possible to observe some isolated initial compounds (Figure 3e), in the lyophilization system (Figure 3f) a new solid phase was formed. In this case, the presence of a homogenous amorphous aggregate was observed.

**Figure 4:**
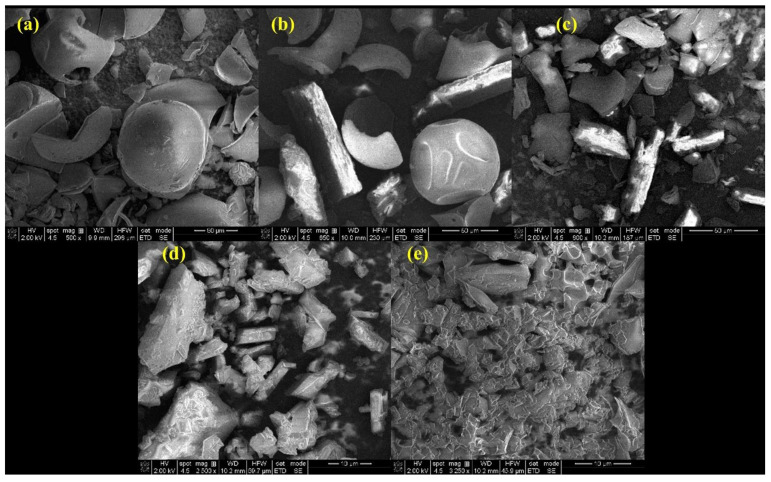
SEM images of (**a**) Me-β-CD, (**b**) AML-Me-β-CD physical mixture, (**c**) AML-Me-β-CD kneading method, (**d**) AML-Me-β-CD coprecipitation method, and (**e**) AML-Me-β-CD lyophilization method.

SEM images of Me-β-CD, the AML-Me-β-CD physical mixture, and AML-Me-β-CD complexes obtained using the kneading, coprecipitation, and lyophilization methods are shown in Figure 4. Me-β-Cd presented fragments of spherical shell morphology (Figure 4a) [39]. By using different synthesis procedures, different morphologies were achieved. The morphology of the AML-Me-β-CD physical mixture (Figure 4b) showed the presence of the two raw materials: a small amount of AML and broken Me-β-CD shells. The chemical methods of preparation changed the morphologies of the final products. If some smaller amlodipine nanoparticles were still present in the system in the case of kneading and coprecipitation processes (Figure 4c,d), the lyophilization method of preparation showed a new homogenous amorphous mixture (Figure 4e).

**Figure 5:**
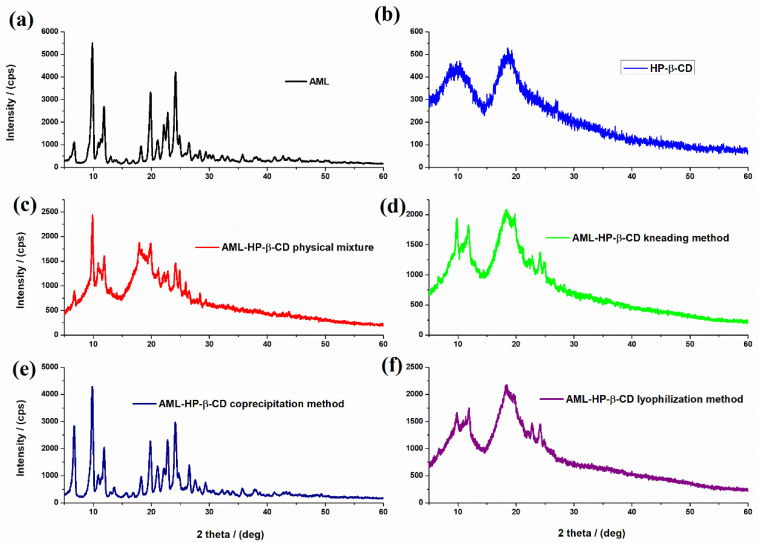
XRD pattern of (**a**) AML, (**b**) HP-β-CD, (**c**) AML-HP-β-CD physical mixture, (**d**) AML-HP-β-CD kneading method, (**e**) AML-HP-β-CD coprecipitation method, and (**f**) AML-HP-β-CD lyophilization method.

**Figure 6:**
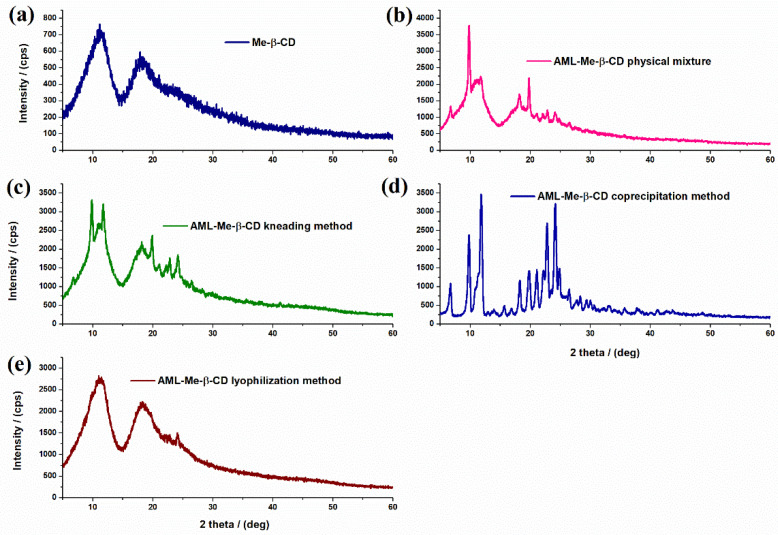
XRD patterns of (**a**) Me-β-CD, (**b**) AML-Me-β-CD physical mixture, (**c**) AML-HP-β-CD kneading method, (**d**) AML-HP-β-CD coprecipitation method, and (**e**) AML-HP-β-CD lyophilization method.

The XRD diffraction method was chosen to evaluate the two cyclodextrins’ complexation. If the complexation process took place and a true inclusion complex was formed, the diffraction pattern of the new formed compounds must have been different for each component [45]. The X-ray diffraction pattern of AML, HP-β-CD, Me-β-CD, the physical mixture compounds, and their complexes are represented in Figure 5 and Figure 6. The XRD diffraction pattern of amlodipine besylate (Figure 5) showed a series of strong diffraction peaks at 2θ = 9.86°, 11.86°, 18.26°, 19.88°, 22.82°, 24.18°, and 26.54°, which indicates its crystalline form [46,47] and corresponds to amlodipine besylate dihydrate (PDF card no. 00-450-2143). The XRD pattern of HP-β-CD (Figure 5) and of Me-β-CD (Figure 6) showed an amorphous phase with broad peaks around 2θ = 9.76° and 18.7° in the case of HP-β-CD, and 2θ = 11.28° and 18.2° in the case of Me-β-CD [39]. Figure 5 shows the XRD pattern recorded for the AML–HP-β-CD physical mixture, the AML–HP-β-CD kneading method, the AML–HP-β-CD coprecipitation method, and the AML–HP-β-CD lyophilization method. In the physical mixture, the characteristic diffraction peaks of AML were presented with a reduction in their intensities, confirming that no interaction appeared in this preparation method. The X-ray diffraction pattern of the compound obtained using the coprecipitation method showed a weak interaction between the two compounds, with the presence of the most important diffraction peaks of AML at lower intensities. On the contrary, the kneading and lyophilization techniques sustained the formation of the inclusion complexes due to the fact that the diffraction AML peaks disappeared, and the new formed compounds showed an amorphous pattern.

Figure 6 shows the XRD pattern of Me-β-CD, the AML-Me-β-CD physical mixture, and the inclusion complexes obtained using the kneading, coprecipitation, and lyophilization methods. In the same way as the AML-HP-β-CD complexes, different synthesis procedures gave different diffraction patterns and in consequence different compounds were obtained. In the case of the physical mixture and for the compound obtained by the kneading and coprecipitation procedures, the presence of some of characteristic diffraction peaks of amlodipine with small intensities proved that the inclusion complex was not formed. The lyophilization method led to obtaining an amorphous compound, and the X-ray diffraction peaks of amlodipine disappeared. The formation of an inclusion complex in this case for AML-Me-β-CD was also confirmed by FTIR analysis.

**Figure 7:**
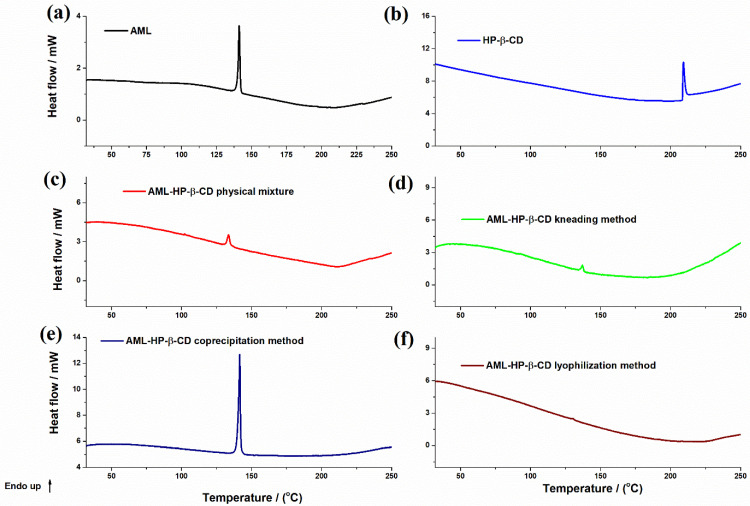
DSC curves of (**a**) AML, (**b**) HP-β-CD, (**c**) AML-HP-β-CD physical mixture, (**d**) AML-HP-β-CD kneading method, (**e**) AML-HP-β-CD coprecipitation method, and (**f**) AML-HP-β-CD lyophilization method.

From the literature, when a guest molecule is included in the cyclodextrin cavity, its melting or sublimation temperature disappears or is shifted to different temperatures [48,49]. Figure 7 shows the DSC thermal curves of AML, HP-β-CD, the AML-HP-β-CD physical mixture, and the AML-HP-β-CD inclusion complexes obtained using the kneading, coprecipitation, and lyophilization methods. Raw materials: AML and HP-β-CD, showed a characteristic endothermal peak corresponding to the melting point: 141.2 °C for AML and 209.2 °C for HP-β-CD [47]. The DSC curves of the physical mixture and inclusion complexes obtained using the kneading and coprecipitation methods showed the presence of the melting peak of AML with lower intensities and shifted at lower temperatures. In the case of the lyophilization method, the melting peak of ALM disappeared, providing evidence of the complexation of the drug into the HP-β-CD cavity [50].

**Figure 8:**
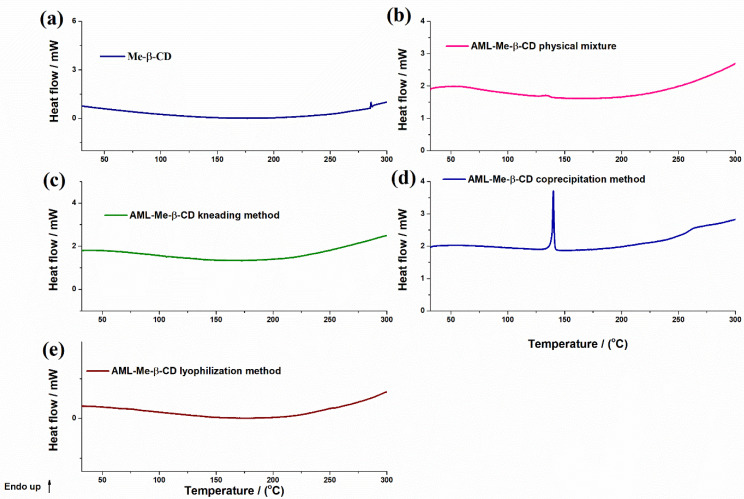
DSC curves of (**a**) Me-β-CD, (**b**) AML-Me-β-CD physical mixture, (**c**) AML-HP-β-CD kneading method, (**d**) AML-HP-β-CD coprecipitation method, and (**e**) AML-HP-β-CD lyophilization method.

Figure 8 displays the DSC curves of Me-β-CD [51], the physical mixture, and the three inclusion complexes. The melting peak of amlodipine could still be distinguished at a lower temperature with different intensities in the physical mixture and the coprecipitation method, proof that the inclusion complex was not formed. The absence of the melting temperature at 140.2 °C was observed in the case of the compound formed by using the kneading method and lyophilization. These assumptions indicate the formation of inclusion complexes in a 1:1 molar ratio using the lyophilization and kneading techniques.

The thermodynamic parameters (*T*_onset_, *T*_max_, *T*_end_) and heat of the thermal effects (Δ*H*) are summarized in Table 3.

All data resulting from physical and chemical analyses led to the assumption that the lyophilization method represents the best route to obtain inclusion complexes of AML with HP-β-CD and AML with Me-β-CD in a 1:1 molar ratio. This indicates the formation of inclusion complexes with only the lyophilization synthesis procedure which was also supported by the FTIR spectra, X-ray diffraction analysis, and DSC data.

### 3.3. Preformulation and Formulation Studies for Orodispersible Tablets Containing AML-HP-β-CD and AML-Me-β-CD Inclusion Complexes

#### Particle Size Distribution

Determining the particle size distribution is an essential step in preformulation studies, as it provides helpful information on the materials’ flowability and compressibility, and also affects the tablets’ pharmacotechnical and disintegration properties [52].

**Figure 9:**
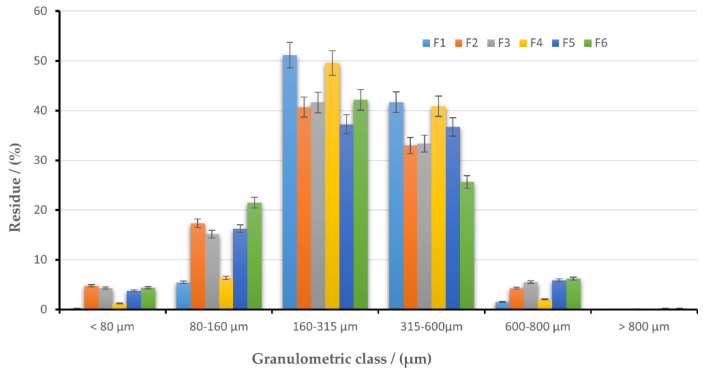
Granulometric analysis of the direct compression blends (F1 is AML-HP-β-CD with silicified microcrystalline cellulose, sodium starch glycolate, and magnesium stearate; F2 is AML-HP-β-CD with F-MELT, sodium starch glycolate, and magnesium stearate; F3 is AML-HP-β-CD with Ludiflash, sodium starch glycolate, and magnesium stearate; F4 is AML-Me-β-CD with silicified microcrystalline cellulose, sodium starch glycolate, and magnesium stearate; F5 is AML-Me-β-CD with F-MELT, sodium starch glycolate, and magnesium stearate; F6 is AML-Me-β-CD with Ludiflash, sodium starch glycolate, and magnesium stearate).

The histogram recorded by representing the particle size distribution of granulometric classes for the six studied materials for direct compression is displayed in Figure 9.

It can be noticed that all particulate systems showed a wide particle size distribution, but differences between the materials are also important to be considered. For all six formulations, most of the particles had sizes fitting in the 160–600 µm range in the first view, predicting similar behavior. Still, F1 and F4 presented a narrow distribution, with low amounts of particles below 160 µm or above 600 µm, whereas the other formulations (F2, F3, F5, and F6) had a broader layout.

In all formulations, particles were forming heterodisperse systems. Although excessive fines lead to the manufacturing of tablets that display defects such as capping and lamination, the presence of some small particles is needed to fill the interstitial spaces between the larger ones [53]. Considering these aspects and the obtained results, F1 and F4, based on silicified microcrystalline cellulose, exhibited a desirable particle size distribution, more suitable than the other formulations for the manufacturing consistency as well as the quality of the tablets.

Particle size distribution also largely influences the segregation phenomenon in powders. A homogenous distribution is generally required; thus, it is preferable to have only small quantities of extreme-size particles [54], and for the studied materials, F1 and F4 are the formulations that contained insignificant proportions of very small or very large particles.

The mechanical properties of powders do not depend only on the fineness or the coarseness of the particles, but also on the interactions between them. Particle size has an impact on the powder rheological parameters, especially on flowability [55]. Flowability is given by the proportion between the cohesive forces and the separation forces.

Table 4 shows the physical and volumetric characteristics of the studied materials.

As expected, certain moisture content was determined in all formulations. With the lyophilization process, amorphous compounds were obtained. Still, a high difference in humidity was observed between materials, proving that not only the preparation technique was influencing the amount of contained water. However, F1 and F4 showed the lowest moisture degree (3.52% for F1 and 4.03% for F4), confirming that neither the cyclodextrin type affected the water uptake. It stands out that the moisture content was dependent on the selected filler excipient, more precisely on the way it increased mixture hygroscopicity. The highest amount of moisture was registered for F6 (7.35%) and F3 (7.11), the formulations that included Ludiflash^®^.

None of the formulations flowed through the 10 mm nozzle, but their flowability could be measured when changing it with the 15 mm nozzle. The behavior is not surprising, as the active ingredients are amorphous compounds, and the blends presented certain moisture and were formed of small-size particles [56]. The registered results confirmed the suitable flowability of F1 and F4, as could be predicted from the previous tests. Only F1 and F4 presented angles of repose below 30, and flow rates of 6.451 g/s for F1 and 5.882 g/s for F4. The flowability was weaker for the materials containing Me-β-CD than the ones with HP-β-CD.

The bulk density did not differ a lot between formulations, but the tapped density had variable values, with a significant increase for the powders based on F-Melt (F2 and F5) and Ludiflash (F3 and F6). The high values of HR (1.29 and 1.24) and CI (22.68 and 19.50) calculated for the formulations that contained the Ludiflash excipient prove the cohesive character of the materials, not desirable for the direct compression process. In addition, F-Melt blends F2 and F5 showed HR and CI values typical for the materials in which the interparticle interactions occur. Meanwhile, the HR and CI values recorded for F1 (1.14 and 12.48) and F4 (1.12 and 14.01), based on silicified microcrystalline cellulose, demonstrate the porous structure of the powders that led to good flowability and compressibility performances.

Based on the assessed physical properties of the powders for direct compression, it was decided to proceed with the study on F1 and F4, as it was expected to achieve a successful manufacturing process and to obtain superior-quality tablets.

### 3.4. Quality Assessment of Orally Dispersible Tablets

**Figure 10:**
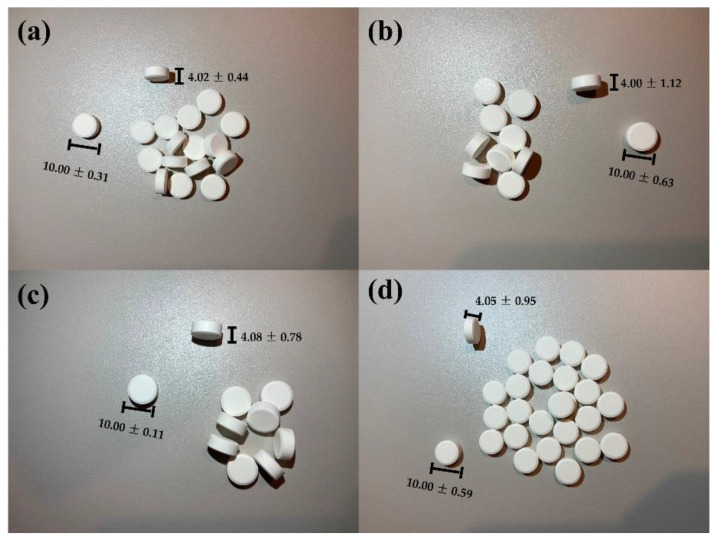
The general appearance of the orodispersible tablet formulations of AML-HP-β-CD: (**a**) F7, (**b**) F8; AML-Me-β-CD: (**c**) F9, (**d**) F10 (F7 is F1 with 30 kN; F8 is F1 with 60 kN; F9 is F4 with 30 kN; F10 is F4 with 60 kN).

The obtained tablets were all round in shape and white, with homogeneous appearances and smooth, flat surfaces (Figure 10).

Table 5 reveals the pharmacotechnical and in vitro properties determined for the four tablet formulations.

All formulations led to tablets with similar sizes, a diameter of 10 mm with a variability below 1% between the tablets of the same batch, and a thickness of around 4 mm with slight differences between the batches depending on the type of cyclodextrin (tablets containing HP-β-CD being more compressed than the ones with Me-β-CD) and on the compression force (the higher pressure of 60 kN inducing advanced compaction). The uniformity of the sizes complies with pharmacopeial requests for all series of tablets [35]. The results are in accordance with the registered values for volumetric characteristics of the powders, especially the tapped density.

The weight uniformity is also in agreement with pharmacopeial standards [35] for all formulations, with insignificant differences between the batches. This proves the accuracy of the formulation and the correct selection of the compression parameters. As the dye filling has the most direct impact on tablet mass, the evaluation of the direct compression materials’ mechanical properties was very important in order to ensure weight uniformity [57]. The lowest weight (347 mg) was presented by F8 containing HP-β-CD and compressed with 30 kN, and the highest mass (350 mg) was registered for F9 based on Me-β-CD and a tableting force of 60 kN.

The hardness varied a lot between the batches, and it decreased in F10 > F8 > F7 > F9 in that order. As expected, the hardness of the tablets was deeply influenced by the used compression force, with an increased value determining a higher tablet tensile strength. The type of cyclodextrin did not much affect the tablets’ mechanical resistance, as the F7 had 44 N and F9 had 42.2 N hardness. Still, even if the tablets exhibit great hardness, they can show unsatisfactory capping or lamination propensity [58]. Consequently, friability must be analyzed in order to establish a tablet’s ability to withstand handling before usage.

The studied tablets manifested an extremely low friability with values in compliance with the pharmacopeial specifications. Both the compression force and the materials’ physical properties influenced the tablets’ friability. The friability of all tablets batches was much lower than the 1% imposed limit, with the highest value of 0.34% for F10.

All formulations showed an excellent disintegration time in the water as well as in the simulated saliva medium, with values in harmony with the imposed regulations for orally dispersible tablets [35]. However, there were differences between the series, depending more on the applied force and less on the type of cyclodextrin. It was noticed that the highest tableting pressure (60 kN) extended the disintegration time by a maximum of 7 s. Meanwhile, the disintegration time varied by a second between the batches containing HP-β-CD and the ones with Me-β-CD.

A variation In the results on different media was also observed, with a surprisingly better disintegration in the simulated saliva medium, for all formulations. The remarkable disintegration of the tablets was achieved by the use of sodium starch glycolate as a superdisintegrant agent and also by the selected filler (silicified microcrystalline cellulose).

Concerning the release performance, all batches proved to have great dissolution rates in agreement with compendial requirements [36]. They displayed values above 85% after 30 min, regardless of the variable formulation or process factors. However, the rates showed significant differences in the function of the dissolution media nature. The highest values were registered under acidic conditions, with more than 98% release for F7, followed by F9 with a 98.11% rate of dissolution. At the same time, in the biorelevant simulated saliva, the amounts dissolved decreased by almost 10% after the same time, with the lowest value of 85.15% displayed by F10.

## 4. Discussion

Flowability, content homogeneity, bioavailability, dissolution, and absorption performance are all closely related to particle size [59]. The direct compression process is also heavily influenced by particle size. Small particles aid with dissolving, but they are also more susceptible to overcompression, resulting in hard tablets with slow disintegration [60].

The results recorded for the flowability and compressibility of the composed powders are in agreement with the registered particle size distribution and their moisture content. As Fu et al. [61] proved, powders with a wide particle size distribution have a lower flowability, but also, as Tunuguntla et al. [62] stated, the influence of extreme-sized particles in small proportions is insignificant. Although F1 and F4 had small-size particles, they were present in inferior amounts than in the other formulations, with this being one of the reasons for their better flowability. Moreover, F3 and F6 had a significant percentage of large particles, above 600 µm. It is clear that the existence of extreme-size particles was due to the selected filler, and Ludiflash presented the widest particle size distribution in comparison to silicified microcrystalline cellulose, which proved to show more homogenous dimensions.

Powder particle size distribution is essential for establishing the critical chemical and physical properties of powders [63].

The powders could be compacted easily due to their high cohesiveness, but the flowability was poor.

The relative increase in moisture content led to an intensification in the cohesion and adhesion forces, thus reducing the flow rates [64]. F2, F3, F5, and F6 contained double the amounts of humidity as F1, and their flowability and compressibility performances were relevantly decreased.

Almost all pharmaceutical powders contain water. When present in low concentrations, it acts as a highly effective plasticizer to promote molecular mobility, and it can accumulate in large amounts in amorphous structures [65]. In the cases of F1 and F4 the moisture ensured the best plasticity, in comparison with the formulations containing F-Melt or Ludiflash excipients.

It was noticed that the Hausner Ratio and Carr Index increased with the increase in moisture content [66], indicating a decreased flowability.

Even F2, F3, F5, and F6 displayed lower values in comparison to F1 and F4; still, they dis exhibit fair to passable flowability. Meantime, F1 and F4 were free-flowing and they had excellent compressibility.

Based on the obtained results for fundamental mechanical properties, F1 and F4 displayed the best plasticity due to the relatively low density, a low moisture content preferable for the direct compression process, a good flowability justified by the uniformity of particle size distribution, and reasons for which we selected these two formulations for the following studies of the research.

It was discovered that powders with an Me-β-CD inclusion complex have lower flowability than powders containing an HP-β-CD inclusion complex, but F-Melt and Ludiflesh also led to diminished flow behavior of the materials.

The tablets’ in vitro performances were significantly influenced by their pharmacotechnical properties.

The uniformity of the tablet sizes and weights between all batches confirmed the accuracy of the formulation that led to a complete filling of the dies due to the optimal flowability of the materials and the consistency of their density without the manifestation of the segregation [67]. It was observed that the dimensional characteristics of the tablets were not influenced by the applied compression force, but by the compaction attributes of the powders.

On one hand, the tablet hardness totally depends on the compression pressure, but on the other hand, the friability does not. There were no significant variations in the hardness of the tablets containing different cyclodextrins, proving that it is not influenced by the nature or structure of the active ingredient.

The influence of the tablets’ hardness on the in vitro disintegration time was obvious. The batches that displayed a stronger mechanical resistance, compressed with a higher force, also showed a longer disintegration time, demonstrating the importance of the process parameters and the correct selection of them. Surprisingly, the type of cyclodextrin did not much impact the disintegration behavior, meaning that AML formed inclusion complexes with similar characteristics with both HP-β-CD and Me-β-CD.

The dissolution performance differed in the function of the dissolution media, being lower in the simulated saliva than in the acidic medium for all formulations. Similar to the other characteristics of the tablets, the rate release did not depend on the type of cyclodextrin, nor on the process parameters. The amlodipine inclusion in the cyclodextrins cavity, as well as the selected excipients, was responsible for this excellent performance.

The tablets had a typical in vitro behavior for orally disintegrated and fast-dissolving tablets [68], proving the proper selection of the inclusion complex-obtaining technique of the tablet excipients and manufacturing process.

## 5. Conclusions

In the present study, the design and evaluation of new orally disintegrating tablets containing amlodipine-hydroxypropyl-β-cyclodextrin and amlodipine–methyl-β-cyclodextrin inclusion complexes were assigned. The complexes were synthesized using three working procedures, namely, the kneading, coprecipitation and lyophilization methods, in a 1:1 molar ratio, and they were compared with a physical mixture. The confirmation of the successful formation of the inclusion complex was carried out using FTIR, SEM, XRD, and DSC analyses. Based on the physical and chemical analysis, the lyophilization method of synthesis was chosen as the best method to obtain a complete inclusion of amlodipine in the cyclodextrin cavity for both used compounds. Subsequently, the preformulation and compression studies of the new orally disintegrated tablets demonstrated that the type of cyclodextrin used has no influence on the dissolution performance of them. The best synthesis procedure and the complete inclusion process are the two factors, besides the choice of proper excipients, that contributed to the final formulation of the tablets.

## Figures and Tables

**Table 1:** The compression material formulation.

Ingredients	Formulation/Amount (%)					
	F1	F2	F3	F4	F5	F6
AML-HP-β-CD	5.39	5.39	5.39	-	-	-
AML-Me-β-CD	-	-	-	6.00	6.00	6.00
PROSOLV^®^ SMCC HD 90-Silicified microcrystalline cellulose	91.75	-	-	91.14	-	-
F-MELT^®^	-	91.75	-	-	91.14	-
Ludiflash^®^	-	-	91.75	-	-	91.14
EXPLOTAB^®^-Sodium starch glycolate	1.86	1.86	1.86	1.86	1.86	1.86
LIGAMED^®^ MF-2-V-Magnesium stearate	1.00	1.00	1.00	1.00	1.00	1.00

**Table 2:** The formulations of the orally dispersible tablets containing AML-HP-β-CD (F7 and F8) and AML-Me-β-CD (F9 and F10) inclusion complexes.

Ingredients	Quantity mg/Tablet				Role in Formulation
	F7 (F1-30 kN)	F8 (F1-60 kN)	F9 (F4-30 kN)	F10 (F4-60 kN)	
Inclusion complex AML-HP-β-CD (1:1)	18.87	18.87	-	-	Active ingredient
Inclusion complex AML-Me-β-CD (1:1)	-	-	21.02	21.02	Active ingredient
PROSOLV^®^ SMCC HD 90-Silicified microcrystalline cellulose	321.13	321.13	318.98	318.98	Filler Binder
EXPLOTAB^®^-Sodium starch glycolate	6.50	6.50	6.50	6.50	Superdisintegrant
LIGAMED^®^ MF-2-V-Magnesium stearate	3.50	3.50	3.50	3.50	Lubricant
TOTAL	350.00	350.00	350.00	350.00	

**Table 3:** Thermodynamic parameters for the thermal processing of the studied samples obtained from DSC measurements.

Sample	Process	*T*_onset_/(°C)	*T*_max_/(°C)	*T*_end_/(°C)	Δ*H/*(Jg^−1^)
AML	Dehydration	82.1	105.9	109.7	21.7
	Melting	140.2	141.2	142.3	80.3
HP-β-CD	Melting	208.6	209.2	210.9	43.2
AML-HP-β-CD physical mixture	Dehydration	101.9	102.2	104.1	0.9
	Melting	131.7	133.6	135.1	13.3
	Decomposition	275.4	285.2	287.1	11.8
AML-HP-β-CD kneading	Dehydration	116.2	118.3	120.0	7.7
	Melting	136.5	137.1	138.2	6.2
	Decomposition	291.2	291.9	292.9	39.0
AML-HP-β-CD coprecipitation	Melting	140.5	141.6	142.4	78.8
AML-HP-β-CD lyophilization	Melting	126.3	130.5	131.3	1.3
	Decomposition	265.4	266.5	268.5	9.5
Me-β-CD	Melting	209.9	210.3	211.1	0.19
	Decomposition	285.5	286.0	286.8	13.9
		287.3	289.1	295.0	21.8
AML-Me-β-CD physical mixture	Melting	129.1	133.6	139.0	6.8
AML-Me-β-CD kneading	Dehydration	66.0	66.4	66.8	0.5
AML-Me-β-CD coprecipitation	Melting	139.0	140.1	141.2	72.7
	Decomposition	235.6	263.9	293.6	41.7
AML-Me-β-CD lyophilization	Dehydration	72.9	76.0	80.7	3.3
	Decomposition	227.9	250.4	250.4	1.1

**Table 4:** Precompression parameters for direct compression powders.

Parameter	Formulation Code					
	F1	F2	F3	F4	F5	F6
Moisture content (%)	3.52 ± 1.31	6.29 ± 1.47	7.11 ± 2.33	4.03 ± 1.57	6.83 ± 2.12	7.35 ± 1.78
Flow time (s) *	9.3 ± 1.06	12.8 ± 1.17	13.1 ± 1.69	10.2 ± 1.31	16.5 ± 1.95	17.2 ± 2.36
Angle of repose (θ degrees) *	26.42 ± 1.26	31.19 ± 2.10	30.89 ± 2.85	27.37 ± 1.48	35.12 ± 2.61	36.44 ± 2.94
Flow rate (g/s) *	6.451	4.687	4.580	5.882	3.636	3.488
Bulk density (g/mL)	0.512	0.577	0.525	0.534	0.563	0.582
Tapped density (g/mL)	0.585	0.682	0.679	0.621	0.681	0.723
Carr Index (CI) (%)	12.48	15.4	22.68	14.01	17.33	19.50
Hausner’s ratio (HR)	1.14	1.18	1.29	1.12	1.20	1.24

* No stirring, nozzle: 15 mm (F1 is AML-HP-β-CD with silicified microcrystalline cellulose, sodium starch glycolate, and magnesium stearate; F2 is AML-HP-β-CD with F-MELT, sodium starch glycolate, and magnesium stearate; F3 is AML-HP-β-CD with Ludiflash, sodium starch glycolate, and magnesium stearate; F4 is AML-Me-β-CD with silicified microcrystalline cellulose, sodium starch glycolate, and magnesium stearate; F5 is AML-Me-β-CD with F-MELT, sodium starch glycolate, and magnesium stearate; F6 is AML-Me-β-CD with Ludiflash, sodium starch glycolate, and magnesium stearate).

**Table 5:** Quality characteristics of the orally dispersible tablets (AML-HP-β-CD: F7 is F1 with 30 kN and F8 is F1 with 60 kN; AML-Me-β-CD: F9 is F4 with 30 kN and F10 is F4 with 60 kN).

Characteristics	Formulation Code			
	F7	F8	F9	F10
Thickness (mm)	4.02 ± 0.44	4.00 ± 1.12	4.08 ± 0.78	4.05 ± 0.95
Diameter (mm)	10 ± 0.31	10 ± 0.63	10 ± 0.11	10 ± 0.59
Mass uniformity	349 ± 1.95	347 ± 2.46	350 ± 1.78	348 ± 3.65
Hardness (N)	44 ± 3.71	57.8 ± 3.55	42.2 ± 3.14	61.2 ± 4.29
Friability (%)	0.03 ± 0.09	0.05 ± 0.12	0.12 ± 0.25	0.34 ± 0.33
*In vitro* disintegration time–in water medium (seconds)	14 ± 1.24	21 ± 2.71	13 ± 1.15	19 ± 1.97
*In vitro* disintegration time–in simulated saliva medium (seconds)	12 ± 3.55	16 ± 2.81	11 ± 3.22	18 ± 4.55
*In vitro* dissolution rate–in acidic medium, after 30 min (%)	98.76 ± 1.45	96.31 ± 2.08	98.11 ± 1.35	97.13 ± 1.92
*In vitro* dissolution rate–in simulated saliva medium, after 30 min (%)	90.18 ± 1.26	88.12 ± 1.84	89.84 ± 1.12	85.15 ± 2.23

## Data Availability

Not applicable.
